# An Immunomodulatory Protein (Ling Zhi-8) from a *Ganoderma lucidum* Induced Acceleration of Wound Healing in Rat Liver Tissues after Monopolar Electrosurgery

**DOI:** 10.1155/2014/916531

**Published:** 2014-05-05

**Authors:** Hao-Jan Lin, Yushan-Sophie Chang, Li-Hsiang Lin, Chiung-Fang Haung, Chia-Yu Wu, Keng-Liang Ou

**Affiliations:** ^1^School of Dentistry, College of Oral Medicine, Taipei Medical University, Taipei 110, Taiwan; ^2^Research Center for Biomedical Implants and Microsurgery Devices, Taipei Medical University, Taipei 110, Taiwan; ^3^Division of Oral Rehabilitation and Center of Pediatric Dentistry, Department of Dentistry, Taipei Medical University Hospital, Taipei 110, Taiwan; ^4^Research Center for Biomedical Devices and Prototyping Production, Taipei Medical University, Taipei 110, Taiwan; ^5^Department of Dentistry, Sijhih Cathay General Hospital, Taipei 221, Taiwan; ^6^School of Dental Technology, College of Oral Medicine, Taipei Medical University, Taipei 110, Taiwan; ^7^Division of Oral and Maxillofacial Surgery, Department of Dentistry, Taipei Medical University Hospital, Taipei, Taiwan; ^8^Graduate Institute of Biomedical Materials and Tissue Engineering, College of Oral Medicine, Taipei Medical University, Taipei 110, Taiwan; ^9^Department of Dentistry, Taipei Medical University-Shuang Ho Hospital, Taipei 235, Taiwan

## Abstract

The purpose of this study was to investigate the effect of an immunomodulatory protein (Ling Zhi-8, LZ-8) on wound healing in rat liver tissues after monopolar electrosurgery. Animals were sacrificed for evaluations at 0, 3, 7, and 28 days postoperatively. It was found that the wound with the LZ-8 treatment significantly increases wound healing. Western blot analysis clearly indicated that the expression of NF-*κ*B was decreased at 3, 7, and 28 days when liver tissues were treated with LZ-8. Moreover, caspase-3 activity of the liver tissue also significantly decreases at 7 and 28 days, respectively. DAPI staining and TUNEL assays revealed that only a minimal dispersion of NF-*κ*B was found on the liver tissue treated with LZ-8 at day 7 as compared with day 3 and tissues without LZ-8 treatment. Similarly, apoptosis was decreased on liver tissues treated with LZ-8 at 7 days when compared to the control (monopolar electrosurgery) tissues. Therefore, the analytical results demonstrated that LZ-8 induced acceleration of wound healing in rat liver tissues after monopolar electrosurgery.

## 1. Introduction

Conventional electrosurgery has been clinically available for more than half a century and widely used as an alternative to the scalpel to reduce blood during surgery. The mechanism of electrosurgical cutting is well defined in all surgical operations areas [1, 2]. However, more attention is focused on the surrounding tissue damage during incision by heat dissipation. The bipolar electrosurgery as an example allows more direct current application but can only be used for hemostasis. The monopolar electrosurgery has been well documented as alternative modalities in oral and maxillofacial surgery, although steel scalpel is considered to be the instrument of choice for surgical incisions [2]. Both monopolar and bipolar surgeries can achieve incision and coagulation of tissue. However, the monopolar mode is more effective than the bipolar for incision or cutting action and possesses distinct advantages over the bipolar mode for this purpose [3]. There are wide varieties of electrode shapes available for monopolar incision than the bipolar. Although incision can be accomplished by both but the bipolar mode is more inefficient and restrictive in application. Bipolar coagulation is slower than monopolar activity [3]. Their slow action is considered a disadvantage for surgical procedures but proves to be of significant benefit for more precise microsurgical procedures, due to their easy usage, wide availability, incision accuracy, and minimal damage to adjacent tissues. The use of steel scalpel in monopolar electrosurgery remains an imperative need for electrosurgery operating system.

The effects of electrosurgery (ES) on histoarchitecture vary depending on the power output [4] and frequency [5] of ES unit, waveform selection [6], and shape and size of active electrode [7]. It is well observed that wound healing for electrosurgery delays through comparative studies for electrosurgical and scalpel wounds [8]. Electrosurgical wounds have more inflammatory responses and tissue destruction but the viability of osteoblasts in both categories shows the same in both kinds of wounds, with no increase in osteoclasts which could indicate no bone resorption occurrences. On the contrary [9], it could be found out that there will be no differences on wound healing between ES and periodontal knives when the gingival resection is shallow. However, there could be intense inflammatory and bone loss in height resulting from bone necrosis during deep resection. Other research study [10] shows that, although there is loss of tissue soon after ES, 70 to 100% of the lost tissue is regained over a period of months. When teeth with cervical amalgam restoration get contact with active electrode tip to simulate, from clinical application report [11], there is no evidence of extensive damage or necrosis of pulp [12]. Macroscopic observation and histological examination of our latest study clearly show that commercial stainless steel needles produce larger injury area than CrN-needles [13]. As reported, slow wound healing found as a result of operations while using both mono- and bipolar electrosurgery devices, an extra therapy is highly recommended to enhance this wound healing after surgery which this paper clearly reports.

“Ling Zhi” is a Chinese term representing combination of spiritual potency and essence of immortality and widely regarded as the “herb of spiritual potency,” symbolizing success, well-being, divine power, and longevity. Among cultivated mushrooms,* G. lucidum* is unique in that its pharmaceutical rather than nutritional value is paramount. A variety of commercial* G. lucidum *products are available in various forms, such as powders, dietary supplements, and tea. These are produced from different parts of the mushroom, including mycelia, spores, and fruit body. Ling Zhi-8 (LZ-8), an immunomodulatory protein isolated from mycelia of* G. lucidum* could be considered as one of the major bioactive substances of its kind [14]. They also contain excellent thermal and acidity stability together with moderate resistivity to alkalinity and dehydration [15]. LZ-8 consists of 110 amino acids and a molecular mass of 12.4 kDa, which is similar to that of the variable region of the immunoglobulin heavy chain [16]. LZ-8 exerts mitogenic activities* in vitro* and has also shown* in vivo* immunomodulatory activities [17]. Ling-Zhi had been widely recommended for specific applications and attributed health benefits for treatment of migraine, hypertension, arthritis, bronchitis, asthma, anorexia, gastritis, hemorrhoids, and diabetes and for the control of blood glucose levels, modulation of the immune system, hepatoprotection, bacteriostasis, and more.

A recent study has well demonstrated that LZ-8 effectively promotes the activation and maturation of immature human dendritic cells via regulating NF-*κ*B and MAPKs path ways [18]. Hitherto, several studies have focused on wound healing actions in different tissues after electrosurgery, inducing inflammatory reaction and reepithelialization, and stimulating angiogenesis. However, there is relatively little information pertaining to wound healing activity and possible mechanism on liver tissues. Hence, the present study was designed to have a three pronged approach: (a) to examine the wound size, injury, fibrosis, and apoptosis in rat model for the liver tissues after monopolar electrosurgery, (b) to ascertain whether LZ-8 protein enhances wound healing for the liver tissues created by surgery and (c) to elucidate the possible mechanism of LZ-8 on wound healing via NF-*κ*B and caspase-3 expressions.

## 2. Materials and Methods

### 2.1. Materials

LZ-8 was provided by Yeastern Biotech Co., Ltd., Taiwan. Anti-NF-*κ*B (MAB3026), caspase-3 (AB1899), *α*-tubulin mAb (MAB1501) monoclonal antibodies (mAbs), horseradish peroxidase (HRP)-conjugated donkey anti-rabbit immunoglobulin G (IgG), and sheep anti-mouse IgG were all from Chemicon, Millipore, Temecula, CA. The nitrocellulose membrane (HybondTM-C Extra, Amersham Biosciences Corp., Hong Kong, China), enhanced chemiluminescence (ECL) Western blotting detection reagent and analysis system (WBLUC0100) were also purchased from Millipore, Temecula, CA. LZ-8 was dissolved in phosphate buffered saline (PBS) and stored at 4°C until the time of usage/application used.

### 2.2. Animals and Surgical Device

Sixteen healthy male Sprague-Dawley rats weighing 200 to 300 grams (g) each, procured from BioLASCO (Taiwan), were used for the experiments. These Sprague-Dawley rats were prepared and maintained under the guidelines for care and usage of laboratory animals at 25°C and exposed to 12 h of dark and 12 h of light. They were fed a standard balanced pellet diet and water ad libitum. The protocols for animal experiments were reviewed and approved by the Institutional Animal Care and Use Committee of Taipei Medical University (LAC-99-0037). Rats were marked to permit individual identification and were kept in their cages for 14 days prior to experimentation to allow for acclimatization to the laboratory conditions. No reusable commercial electrosurgical monopolar tip (ERBE, number 20191-377, USA; needle type, stainless steel 304) was used.

### 2.3. Surgical Procedure

General anesthesia was induced with inhaled isoflurane for the animals. The operative site was cleansed and draped in a sterile fashion. Conventional stainless steel (SS) needle electrodes were used with an electrosurgical unit. The liver was exposed through midline laparotomy with a retractor to avoid injury to the liver. An unmodified SS-needle was inserted to the liver lobe (3 mm in depth) to create an anterior lesion, using a fixed power setting (20 W) with a fixed activation time (3 seconds). The power setting and activation time were commonly used parameters and were based on previous study settings. For LZ-8 protein group, 10 *μ*L of 1 mg/mL LZ-8 protein solution was pipetted and dropped on the wound surface. For control group and experiment group, 10 *μ*L of normal saline was pipetted and dropped on the wound surface.

### 2.4. Weight of Sticking Tissue

The weights of each SS-needle were recorded perioperatively, and the weight of sticking tissue was calculated as the weight of needle after operation minus the weight of needle before operation.

### 2.5. Thermography

The effects of the SS-needles on temperatures across the live lobe surface were determined from thermographs obtained with a thermal-imaging infrared camera (Advanced Thermo TVS-500EX, NEC Avio Technologies, Tokyo, Japan). The highest temperature was recorded for every test. After recording the thermograph, the surrounding soft tissues were carefully repaired and the surgical wound was closed in layers. No anti-inflammatory regimen was used after surgery.

### 2.6. Histological Examination to Detect Injury Area

Histopathological studies were performed to provide structural evidence of mediated tissue injury and protective effect of LZ-8. These animals were sacrificed on days 0, 3, 7, and 28 after operation, respectively. Tissues were then cut into pieces of desired size and fixed in Bouin's fluid fixative immediately after autopsy. Fixation was carried out at room temperature for 24 hrs, after which these fix tissues were transferred to 70% alcohol. Several changes of 70% alcohol were given until the yellow colour disappeared from the tissues. The tissues were then dehydrated by passing through ascending grades of alcohol (30%, 50%, 70%, 90%, and 100%), cleared in methyl salicylate and infiltrated with wax at 57°C. The clear tissues were embedded in the paraffin. Conventional techniques of paraffin wax sectioning were used for histological studies [19]. Paraffin wax sections of 6 to 8 *μ*m thickness were cut using a rotary microtome (Leica, Germany). The sections were further stained with hematoxylin and eosin (H&E) and Masson's trichrome staining and then washed in 90% alcohol for few seconds. Subsequently, the stained sections were dehydrated in 100% alcohol, cleared in xylene, and mounted in DPX mountant. The stained slides were observed in a Carl Zeiss (Germany) Axio 2 Plus research microscope. Images were captured through a CCD camera (BX51, Olympus, Japan) in a computer and processed using Carl Zeiss Axiovision software. The total injury areas caused by the SS-needle are measured using image analysis software (SPOT basic software, SPOT imaging solutions, MI, USA). In addition, different histological features were observed on the injury site. Therefore, the injured area was further divided into different classifications to demonstrate the injury caused by thermal spread, and the area of every portion was measured.

### 2.7. Western Blot Analysis

The retrieved liver tissues were homogenized and lysed in an ice-cold protein lysis buffer and centrifuged at 13000 rpm for 15 minutes at 4°C. Protein concentrations of the lysates were determined using a Bio-Rad protein assay (Bio-Rad Laboratories, Hercules, CA, USA). The lysates (20 *μ*g/lane) were resolved by sodium dodecyl sulfate polyacrylamide gel electrophoresis (SDS-PAGE) in 10% gels and electroblotted onto a nitrocellulose membrane (HybondTM-C Extra, Amersham Biosciences Corp., Hong Kong, China). The membrane was blocked for 30 minutes at room temperature in 3% nonfat milk Tris-buffered saline with 0.05% Tween 20, and then incubated overnight at 4°C with the indicated primary antibodies against NF-*κ*B (1 : 500, MAB3026, Chemicon, Millipore, Temecula, CA) or caspase-3 (1 : 200, AB1899, Millipore, Temecula, CA), and then incubated with horseradish peroxidase-conjugated secondary antibodies for 2 hours at room temperature. After washing, the membrane was developed using chemiluminescent substrates (WBLUC0100, Millipore, Temecula, CA). Densitometric analysis of the gels was performed using Gel-Pro analyzer software, and the results were expressed relative to the density of *β*-actin (1 : 500, MAB1501, Chemicon, Millipore, Temecula, CA).

### 2.8. Immunofluorescence Staining and TUNEL Staining

For immunofluorescence staining, liver tissues were embedded in Cryomatrix (Thermo Shandon, Pittsburgh, PA, USA) and stored at −80°C. Cryostat sections (5–7 mm thickness) were fixed in a mixture of methanol and acetone (1 : 1) at −20°C for 5 minutes and incubate in 1% Triton X-100 for another 5 minutes. Nonspecific activity was blocked by incubating the sections with 10% horse serum in a buffer containing 0.02% Tween 20 in phosphate-buffered saline for 30 minutes. Sections were then incubated overnight with primary anti-Caspase-3 at 4°C. Antigen localization was indicated by a Cy5 dye (red fluorescence, 111-176-003, Jackson Immune Research, West Grove, PA). Finally, the samples were stained with 4′,6-diamidino-2-phenyl-indole (DAPI, sc-3598, Santa Cruz Biotechnology, CA) for 7 minutes and examined using fluorescent microscope. Normal liver tissues obtained from healthy rats undergo identical procedures. Negative controls were processed identically, except that the primary antibodies were replaced with IgG. An* in situ *cell death detection kit (terminal deoxynucleotidyl transferase dUTP nick end labeling, TUNEL, 11684795910, Roche, Mannheim, Germany) was used to detect apoptotic cells, and the apoptotic cells were indicated by a green fluorescent FITC fluorochrome.

### 2.9. Statistical Analysis

Data were expressed as mean ± standard error of the mean (SEM). Data were analyzed using analysis of variance (ANOVA). All statistical analyses were performed using SPSS version 12.0 (SPSS, Inc, Chicago, IL). Values of *P* < 0.05 were considered significant.

## 3. Results

### 3.1. Stimulatory Effects of LZ-8 on Wound Healing in Rat Liver Tissues after Monopolar Electrosurgery

**Figure 1:**
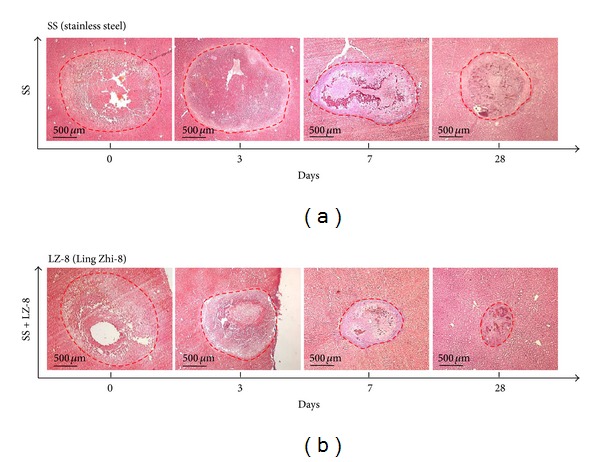
The wound healing effects on wounds in rat liver tissues after monopolar electrosurgery for different healing days (H&E staining): (a) SS group and (b) SS + LZ-8 group.

In generally, bleeding and healing could easily be reduced when tissues are subjected to electrosurgery. To determine whether LZ-8 could enhance wound healing on liver tissues after surgery, we compared the wound size after monopolar electrosurgery and the operated tissues that had been treated with LZ-8 for 0, 3, 7, and 28 days. The observed results of H&E stained section of liver tissues show that a significant reduced of wound size treated with LZ-8 (3, 7, and 28 days) dependently relative to the normal (untreated) rats as shown in Figure 1. Moreover, the mean wound sizes for these liver tissues were more prominently reduced in tissues that were treated with LZ-8 at 28 days when compared with untreated groups and with day 3 and day 7. The results clearly indicated that treatment with LZ-8 enhances wounds healing faster after surgery in rat liver tissues.

### 3.2. Effects of LZ-8 on the Site of Thermal Damage, Injury, and Wounds of Liver Tissues after Surgery

**Figure 2:**
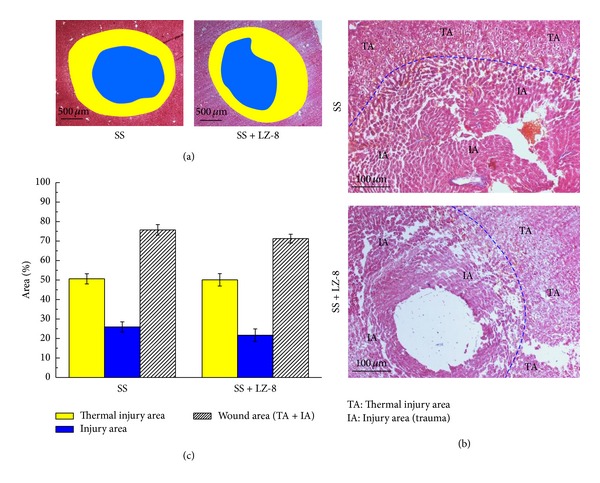
The wound healing effects on wounds in rat liver tissues after monopolar electrosurgery for 0 days (Masson's trichrome staining): (a) wound area, (b) the site of thermal injury area and injury area, and (c) percentage of each area. Data are expressed as the means ± SEM (*n* = 5). No significant difference in the thermal injury area, injury area, and wound area at day 0.

**Figure 3:**
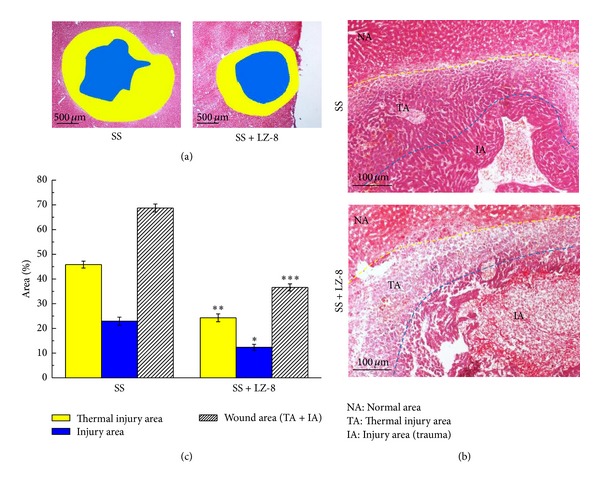
The wound healing effects on wounds in rat liver tissues after monopolar electrosurgery for 3 days (Masson's trichrome staining): (a) wound area, (b) the site of thermal injury area, injury area, and normal area, and (c) percentage of each area. Data are expressed as the means ± SEM (*n* = 5). _ _*P < 0.05, _ _**P < 0.01, _ _***P < 0.001 compared with SS group.

Figure 2 shows histological sections of monopolar SS on the operated liver tissues and tissues subjected to LZ-8 treatment (SS + LZ-8) for 0 days (Masson's trichrome staining). Clearly, there was no significant difference in the total injury areas caused by the SS and SS + LZ-8 treatments. Moreover, the injury area and thermal injury area in SS group were also similar to those of SS + LZ-8 group at day 0. However, the results demonstrated that damage of liver tissues in the form of injury, thermal injury, and wound was negligible in SS group for 3 days (Figure 3) when compared to 0 days (Figure 2). Interestingly, the effect of injury, thermal injury, and wound of liver tissues was significantly reduced in SS + LZ-8 group for 3 days. It was found that the injury area (*P* < 0.05), thermal injury area (*P* < 0.01), and wound area (*P* < 0.001) of SS + LZ-8 group were reduced more significantly than those of SS group at day 3. Furthermore, Masson's trichrome staining was used to examine the formation of fibrotic tissue. No fibrotic tissue was evident for either group at day 0 or day 3.

### 3.3. Effects of LZ-8 on the Areas of Fibrosis, Injury, Apoptosis, and Wounds of Liver Tissues after 7 Days of Surgery

**Figure 4:**
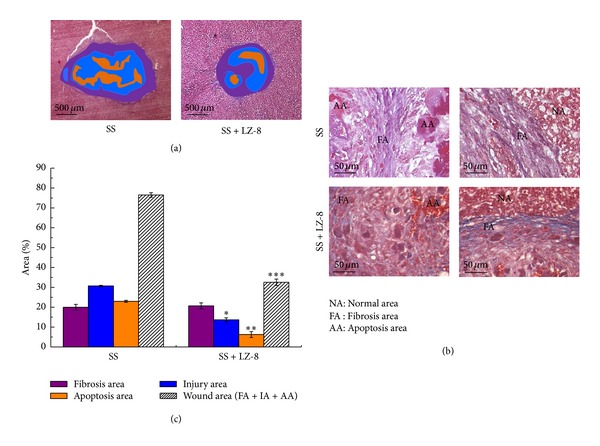
The wound healing effects on wounds in rat liver tissues after monopolar electrosurgery for 7 days (Masson's trichrome staining): (a) wound area, (b) the site of apoptosis area, fibrosis area, and normal area, and (c) percentage of each area. Data are expressed as the means ± SEM (*n* = 5). _ _*P < 0.05, _ _**P < 0.01, _ _***P < 0.001 compared with SS group.

A further study was performed to determine whether LZ-8 could enhance the areas of fibrosis, reduce injury and wounds, and inhibit apoptosis of monopolar SS induced liver incision in terms of withdrawing the above observed injuries. The results clearly confirmed that injury, apoptosis, and wound of liver tissues in SS operated groups that had been treated with LZ-8 for 7 days were significantly alleviated when compared with SS operated groups without LZ-8 treatment (Figure 4). Nevertheless, the areas of fibrosis and numbers of cell death in liver tissues for treated rats with LZ-8 were comparatively higher than those of SS operated rats. Thus, the results indicate that LZ-8 treatment could not affect the reduction of liver fibrosis and more effective on the inhibition of apoptosis which are caused by surgery.

### 3.4. Effects of LZ-8 on the Areas of Fibrosis, Injury, Apoptosis, and Wounds of Liver Tissues after 28 Days of Surgery

**Figure 5:**
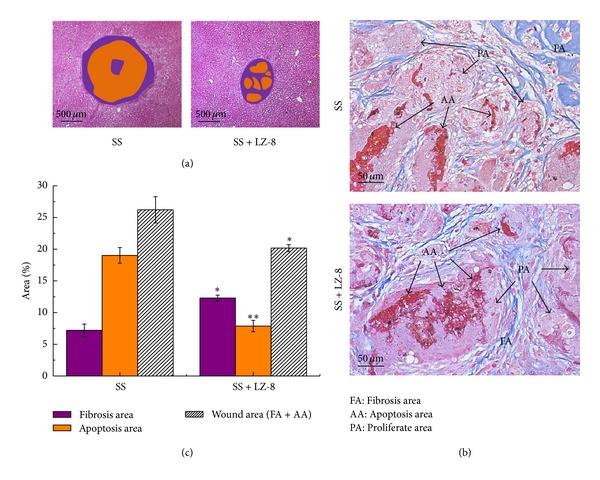
The wound healing effects on wounds in rat liver tissues after monopolar electrosurgery for 28 days (Masson's trichrome staining): (a) wound area, (b) the site of apoptosis area, fibrosis area, and proliferate area, and (c) percentage of each area. Data are expressed as the means ± SEM (*n* = 5). _ _*P < 0.05, _ _**P < 0.01, _ _***P < 0.001 compared with SS group.

Since differential observations were found on the areas of fibrosis and apoptosis in liver tissues of LZ-8 supplemented rats that have already been operated with SS for 7 days, we further extended the duration of treatment up to 28 days (Figure 5) to confirm the hypothesis. Our analysis obviously exhibits further evidence that the effect of LZ-8 was similar to that of 7-days treatment.

### 3.5. Does LZ-8 Enhance Wound Healing in SS Operated Liver Tissues via Inhibiting the Expression of NF-*κ*B and Caspase-3?

**Figure 6:**
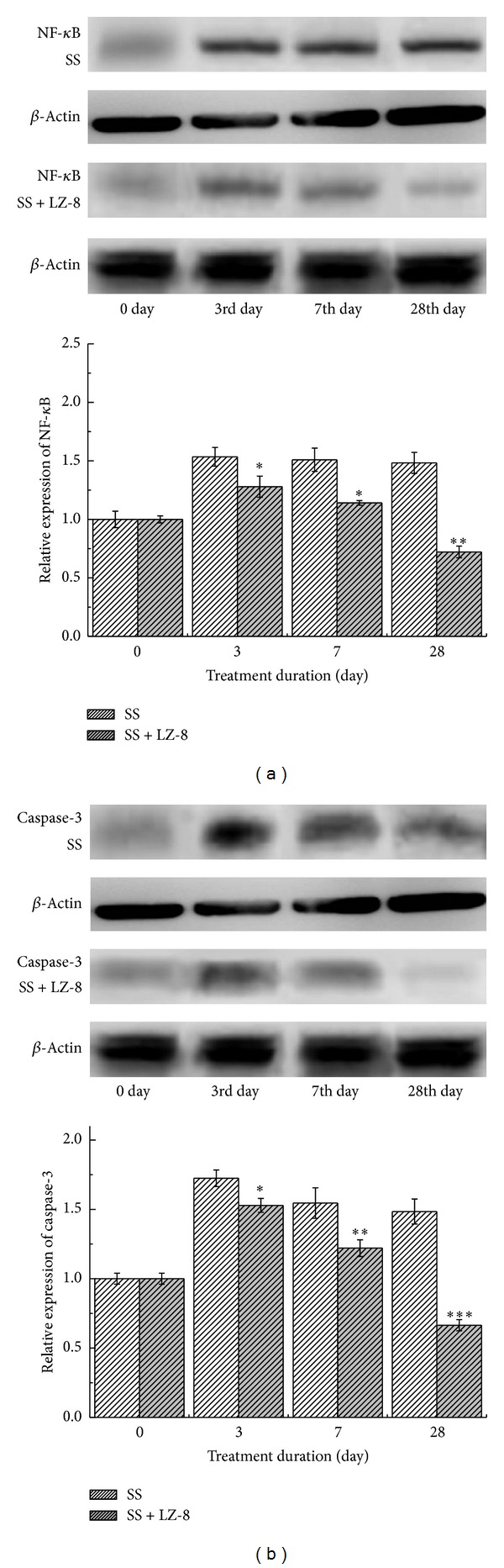
The Western blot assay results from rats liver treated with SS group and SS + LZ-8 group after different healing days: (a) expressions of NF-*κ*B and (b) expressions of caspase-3. Data are expressed as the means ± SEM (*n* = 5). _ _*P < 0.05, _ _**P < 0.01, _ _***P < 0.001 compared with SS group.

It is well known that the angiogenesis is essential in wound healing. Various studies have demonstrated the inhibition of angiogenesis by the blockage of NF-*κ*B. A recent study has also suggested that caspase-3 plays an important role during skin wound healing [20]. Thus, we performed this study to determine whether the wound healing action of LZ-8 was due to the effect on inhibiting of NF-*κ*B and caspase-3 expression, the tissue levels of their protein were examined by Western blot analysis. As shown in Figure 6(a), liver tissues exposed to electrosurgery with SS for 3 days could slightly induce the expression of NF-*κ*B and it gradually increased up to 28 days. Interestingly, liver tissues further treated with LZ-8 significantly reduced the ratio of NF-*κ*B expression in a duration dependent manner (3 to 28 days). A similar result was observed on the expression of caspase-3. Therefore, the results can suggest that treatment of liver tissues after surgery with LZ-8 was found to lower the mean NF-*κ*B and caspase-3 expressions, so that it approaches wound healing property, presumably by limiting NF-*κ*B and caspase-3 expression in liver tissues. These results were further confirmed and shown as in Figure 6(b).

### 3.6. LZ-8 Inhibits NF-*κ*B Nuclear Translocation in SS Operated Liver Tissues

**Figure 7:**
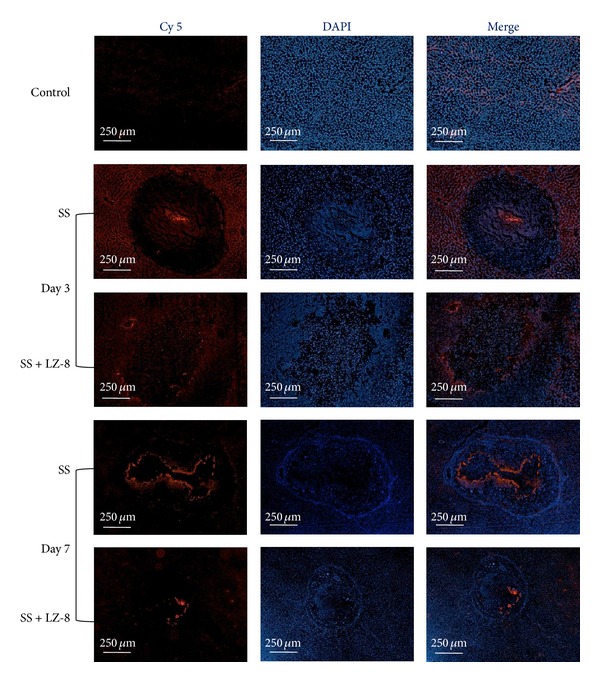
The immunofluorescence staining results from rats liver treated with SS group and SS + LZ-8 group after different healing days.

The ability of LZ-8 to inhibit NF-*κ*B dispersion was determined by red fluorescence (Cy5) intensity analysis of immunolabelled antibodies directed against NF-*κ*B and Hoechst reagent to simultaneously identify nuclei (Figure 7). The intensity values of NF-*κ*B red fluorescence in the nucleus of SS operated tissues that were treated with LZ-8 for 3 days were found to be lesser as compared to SS operated liver tissues without LZ-8 treatment. Interestingly, the presence of LZ-8 in SS treated liver tissues for 7 days reached a maximum inhibition of nuclear fluorescence of NF-*κ*B than that of treated tissues with SS alone. These results can obviously indicate that LZ-8 plays a major role in inhibiting of NF-*κ*B nuclear translocation in liver tissues during the process of wound healing after electrosurgery.

### 3.7. LZ-8 Reduces Apoptotic Cell Death in Liver Tissues during Wound Healing after Electrosurgery

**Figure 8:**
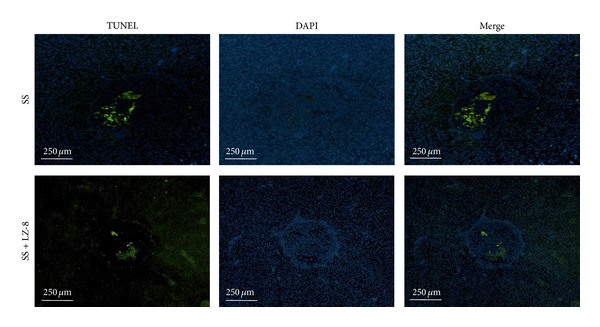
The TUNEL staining assay results from rats liver treated with SS group and SS + LZ-8 group after 7 days of healing.

Extensive fragmentation of nuclear DNA that generates a large number of DNA double-strand breaks is one of the most characteristic events of apoptosis. An assay that relies on detection of DNA strand breaks (DSBs)* in situ* by labeling them with fluorochromes has been developed to identify and quantify apoptotic cells by fluorescence microscopy or cytometry. The assay is commonly called TUNEL, the acronym of Terminal deoxynucleotidyl transferase-mediated d-UTP Nick End Labeling. In this study, a very clear TUNEL-positive cells were observed in rats hepatic cells after 7 days of electrosurgery (Figure 8). However, the number or TUNEL expression continued to decrease more prominently in LZ-8 treated hepatic cells that had been exposed with monopolar electrosurgery for 7 days. The results obtained from this study demonstrate that a slight apoptosis occurs in hepatic cells after electrosurgery which was as a result of LZ-8 inhibition and provide evidence for high level of angiogenesis arises during wound healing process.

## 4. Discussion

The purpose of this study was to determine the effect of the LZ-8, an immunomodulatory protein, derived from* G. lucidum*, when it is used for wound healing on hepatic tissues and to compare this with monopolar electrosurgery operated liver tissues, on the wounds size, fibrosis, thermal injury, and apoptosis. The results of this study show that LZ-8 enhances wound healing actions in hepatic tissues after electrosurgery. Importantly, this study has identified that NF-*κ*B and caspase-3 activation are associated with LZ-8 on enhancing wound healing after surgery. Purpose of electrical energy or coagulation of bleeding vessels onto tissue is frequently used in surgery. Coagulation techniques such as monopolar and laser techniques are commonly used in different surgical disciplines [21, 22].

Monopolar and bipolar coagulation effects on brain tissue were reported and the results of experimental trials were compared [23, 24]. They observed less trauma after bipolar coagulation compared with monopolar technique. A study has demonstrated that bipolar mode of stimulation claimed to be another paradigm of a potential hazard compared with monopolar stimulation. Temel et al. [25] observed brain damage after bipolar, but not monopolar and stimulation in rats. However, monophasic pulses were used, which might have created larger lesions in a bipolar configuration. Monopolar electrosurgery causes sparking, current spread, and thermal damage in the tissues because of excessive generation of heat. These problems will induce deleterious effects to the tissue in proximity to the operative site. In addition, when comparing monopolar electrosurgery and the scalpel incision, electrosurgery produces more tissue alteration and histological thermal damage when a lateral heat was increased by the low frequency radio wave of 0.5 to 2.9 MHz [26]. Consequently, the resultant effect of monopolar systems is excessive tissue damage and delayed healing. Therefore, additional drug supplements or therapy need to be required to accelerate the wound healing action on tissues after electrosurgery.

A new immunomodulatory protein, known as LZ-8, was isolated from the mycelia of* G. lucidum *and characterized by chromatographic/electrophoretic techniques [17]. Both* in vivo* immunomodulation activity and* in vitro* mitogenic activity of LZ-8 have been reported by Kino et al. [14]. Previous studies have also demonstrated that topical application of oils improves some parameters of wound healing such as stimulation of connective tissue fiber [27], reduction in the thickness of the necrotic cell layer edge around the wound [28], and acceleration of wound closure [29, 30]. Due to excellent thermal and acid stability together with a moderate resistance to alkali and dehydration, LZ-8 is appropriate to apply tissues after surgery even with a mild thermal heat. In this study, this is the first and novel results that LZ-8 accelerates the reduction of the wound size, thermal injury, and increase fibrosis area in rat liver after surgery and it indicates that LZ-8 has potent ability to enhance the effect of wound healing. In a latest study, we have also demonstrated that the total areas of liver injury, lateral thermal injury, and fibrotic tissues were significantly higher in SS treated hepatic tissues than that of CrN-coated needles that had been used for surgery [13].

NF-*κ*B activation plays a role in the regulation of more than 400 gene product expressions associated with inflammation, cell survival, proliferation, invasion, and angiogenesis [31, 32]. NF-*κ*B normally binds to I*κ*B*α*, which impedes NF-*κ*B nuclear translocation from the cytoplasm to the nucleus. NF-*κ*B is reported to be a vital link between hepatic injury, fibrosis, and even hepatocellular carcinoma [33]. NF-*κ*B enters the nucleus, with the degradation of I*κ*B and activates the expression of specific genes that induce the apoptosis cascade [34]. Once the cells are exposed to inflammatory stimuli, including LPS and TNF-*α*, I*κ*B*α* is phosphorylated, leading to I*κ*B*α* degradation and nuclear translocation of NF-*κ*B. In our latest study, it was noticed that liver tissues that had been operated with SS-needles secreted higher levels of NF-*κ*B expression and its nuclear translocation than that of CrN operated tissues [13]. Fibrosis is an important component of advanced chronic inflammatory liver disease. Although the changes in hepatic structures that occur in liver cirrhosis have been described in detail, the pathogenesis of fibrosis in during electrosurgery process is largely unknown. Our latest report indicates that monopolar elecrtrosurgery-induced hepatic fibrosis after repeated liver injury and the mechanisms are mainly based on the upregulation of NF-*κ*B and caspase-3 expression in the liver tissues [13].

In this study, we thus evaluated whether LZ-8 had any effect on nuclear translocation of NF-*κ*B during monopolar electrosurgery and the results found for the first time that LZ-8 inhibits nuclear translocation of NF-*κ*B in hepatic cells after 3 and 7 days of monopolar electrosurgery. Yates and Rayner [35] studied the effects of fatty acid supplementation on activation of NF-*κ*B and AP-1 after a skin injury, since these transcription factors are highly associated with wound healing. They have also reported a novel finding which indicates that wounding of normal human keratinocytes in culture causes NF-*κ*B translocation. The relevance of NF-*κ*B activation to wound healing is suggested by the results that HeNe laser treatment, a process shown to accelerate healing in a model of wounding [36], promoted the translocation of NF-*κ*B. A previous study also proved that a ganoderma immunomodulatory protein, cloned from* Ganoderma microsporum* holds anti-invasive and anti-inflammatory activities in TNF-*α* induced human alveolar epithelial A549 cells via inhibition of the I*κ*B*α* and NF-*κ*B pathway [37].

Apoptosis in wound healing is a promising issue. There has been some evaluation of chronic diabetic wounds in mice that suggests that there is increased cellular apoptosis likely due to hyperglycemia, which produces reactive oxygen species that mediate mitochondrial release of cytochrome c and activate caspase-3 [37, 38]. Apoptosis is also involved in the regulation of collagen synthesis and degradation within wounds through regulation of fibroblast numbers and collagenase activity [39]. Recent work in another mouse model suggests that hypertrophic scars demonstrate decreased rates of apoptosis, with decreased expression of cleaved caspase-3. Over expression of various caspase family members induces apoptosis in cultured mammalian cells [40]. Among the cysteine proteases, caspase-3 is believed to be one of the most commonly involved in the execution of apoptosis in various cell types [41]. The assay of TUNEL staining can identify the apoptotic cells via DNA fragmentation by labeling the terminal ends of nucleic acids [42, 43]. From our latest study of TUNEL assay, it can be clearly found that the apoptosis event was more significant in SS-needle operated liver tissues [13]. In this study, TUNEL staining provides direct evidence of apoptosis in SS operated liver tissues after 7 days. Both the activation of caspase-3 and the induction of apoptotic cell death could be suppressed by the addition of antioxidants in the cultures [44]. It has also been demonstrated that LZ-8 is able to effectively promote the activation and maturation of immature dendritic cells via the nuclear factor-kappaB and mitogen-activated protein kinase pathways [18]. Moreover, the present study observed the effects of (i) inhibiting expression and nuclear translocation of NF-*κ*B and (ii) inhibiting expression of caspase-3 and reduction of apoptotic cell death by LZ-8 in liver tissues after electrosurgery which indicates that the wound healing property of LZ-8 may possibly occur through inhibition of transcription factor NF-*κ*B and apoptotic factor caspase-3.

## 5. Conclusion

Under the same conditions, LZ-8 treatment potentiates on reducing wound size and thermal injury. An increasing fibrosis and reducing apoptosis were found in LZ-8 treated liver tissues during electrosurgery. LZ-8 treatment significantly inhibits NF-*κ*B and caspase-3 expressions and also reduces NF-*κ*B nuclear translocation and apoptotic cell death. The results generated from this study suggest that LZ-8 has potent effects on increasing wound healing action against the electrosurgical induced liver injury via the possible mechanism of inhibiting NF-*κ*B and caspase-3 expressions.

## Supplementary Material

Original image of Figure 2(a).
Original image of Figure 3(a).
Original image of Figure 4(a).
