# Supplementary material for: An Immunomodulatory Protein (Ling Zhi-8) from a Ganoderma lucidum Induced Acceleration of Wound Healing in Rat Liver Tissues after Monopolar Electrosurgery
Source: Evid Based Complement Alternat Med. 2014 May 5;2014:916531. doi: 10.1155/2014/916531 (PMC4026841; doi:10.1155/2014/916531)
Supplement: Supplementary file 1 — Original image of Figure 2(a). Original image of Figure 3(a). Original image of Figure 4(a). [file 916531.f1.docx]

**Original image of Fig. 2(a)**

**Original image of Fig. 3(a)**

**Original image of Fig. 4(a)**
